# Maternal effects as drivers of sibling competition in a parent–offspring conflict context? An experimental test

**DOI:** 10.1002/ece3.1777

**Published:** 2016-05-03

**Authors:** Thomas Merkling, Charlotte Perrot, Fabrice Helfenstein, Jean‐Baptiste Ferdy, Laurent Gaillard, Emilie Lefol, Emmanuelle Voisin, Scott A. Hatch, Etienne Danchin, Pierrick Blanchard

**Affiliations:** ^1^ CNRS ENFA UMR5174 EDB (Laboratoire Évolution & Diversité Biologique) Université Toulouse 3 Paul Sabatier 118 route de Narbonne F‐31062 Toulouse France; ^2^ Institute of Biology University of Neuchâtel Rue Emile‐Argand 11 CH‐2000 Neuchâtel Switzerland; ^3^ Institute for Seabird Research and Conservation 12850 Mountain Place Anchorage Alaska 99516; ^4^ Present address: CEFE UMR 5175 CNRS EPHE Université de Montpellier Université Paul‐Valéry Montpellier 1919 Route de Mende 34293 Montpellier Cedex 5 France; ^5^ Present address: Centre de recherche de La Tour du Valat Le Sambuc 13200 Arles France

**Keywords:** Brood reduction, benefit/cost ratio, facultative siblicide, food availability, phenotypic plasticity, yolk testosterone

## Abstract

Maternal effects occur when the mother's phenotype influences her offspring's phenotype. In birds, differential allocation in egg yolk components can allow mothers to compensate for the competitive disadvantage of junior chicks. We hypothesize that the parent–older chick conflict peaks at intermediate conditions: parents benefit from the younger chick(s) survival, but its death benefits the older chick in terms of growth and survival. We thus expect maternal compensation to follow a bell‐shaped pattern in relation to environmental conditions. We studied a black‐legged kittiwake (*Rissa tridactyla*) population where previous results revealed increased allocation of yolk testosterone in younger as compared to older chicks in intermediate conditions, in line with our theoretical framework. We therefore predicted a maternally induced increase in aggressiveness, growth, and survival for younger chicks born in intermediate environmental conditions. Controlling for parental effects and chick sex, we manipulated food availability before egg laying to create a situation with intermediate (Unfed group) and good (Fed group) environmental conditions. Within each feeding treatment, we further created experimental broods where the natural hatching order was reversed to maximize our chances to observe an effect of feeding treatment on the younger chicks' aggressiveness. As predicted, we found that chick aggressiveness was higher in younger chicks born from the Unfed group (i.e., in intermediate environmental conditions), but only when they were put in a senior position, in reversed broods. Predictions on growth and survival were not confirmed. Mothers thus seem to favor the competitiveness of their younger chick in intermediate conditions via egg yolk components, but our study also suggests that hatching asynchrony need to be small for maternal compensation to be efficient. We emphasize the need for further studies investigating other chick behaviors (e.g., begging) and focusing on the relative role of different yolk components in shaping parent–offspring conflict over sibling competition.

## Introduction

Mothers, or the environment they experience before reproduction, are known to influence their offspring's phenotype and fitness beyond the direct effect of their genes (Mousseau and Fox 1998; Marshall and Uller 2007; Wolf and Wade 2009). In the last decades, these so‐called maternal effects have been studied in a wide range of taxa such as plants (reviewed in Gutterman 2000), insects (reviewed in Mousseau and Dingle 1991), mammals (reviewed in Maestripieri and Mateo 2009), and reptiles (e.g., De Fraipont et al. 2000). However, bird studies on the role of various egg yolk components on the resulting chick phenotype have probably been the most numerous. Carotenoids (e.g., Saino et al. 2003), antibodies (e.g., Hasselquist and Nilsson 2009), and hormones (e.g., Groothuis et al. 2005b) transferred in yolk by mothers have been shown to positively influence the immune system, growth, and behavior of chicks during the rearing period and even beyond (Groothuis and Schwabl 2008). Evidence suggests that the cost to mothers of bestowing egg yolk with such components is low (Groothuis et al. 2005b; Uller 2008). Hence, maternal effects are supposed to have evolved as a way for mothers to increase their fitness according to the prevailing environment (Marshall and Uller 2007; Müller et al. 2007), leading, for example, to increased chick competitiveness and growth (e.g., Eising et al. 2001).

Chicks often hatch asynchronously and last‐hatched, younger chicks (hereafter junior chicks) typically have a size and competitive disadvantage as compared to their older siblings (hereafter senior chicks), thereby making them more vulnerable (Mock and Parker 1997). By differentially provisioning last‐laid eggs with more yolk components, mothers could enable their junior chicks to compensate for their competitive disadvantage, thus preventing brood reduction (Müller and Groothuis 2013).

However, maximization of maternal fitness may not necessarily arise by strongly compensating for the disadvantage of junior chicks in all situations. External factors such as food availability are expected to influence the benefits and costs of such a strategy, as illustrated by studies showing effects of food availability on yolk androgens (e.g., Verboven et al. 2003; Vergauwen et al. 2012). In line with this, difference in yolk testosterone levels between the second and the first egg of black‐legged kittiwakes (*Rissa tridactyla*) has been reported to be larger (i.e., higher levels in second as compared to first‐laid egg) when food availability was intermediate than when it was low or high (Benowitz‐Fredericks et al. 2013). Because yolk testosterone can increase growth, begging (Groothuis et al. 2005b), and aggressiveness (Müller et al. 2012), these results suggest that mothers favor their junior chick(s)' competitiveness and thereby survival, especially when conditions are intermediate.

**Figure 1:**
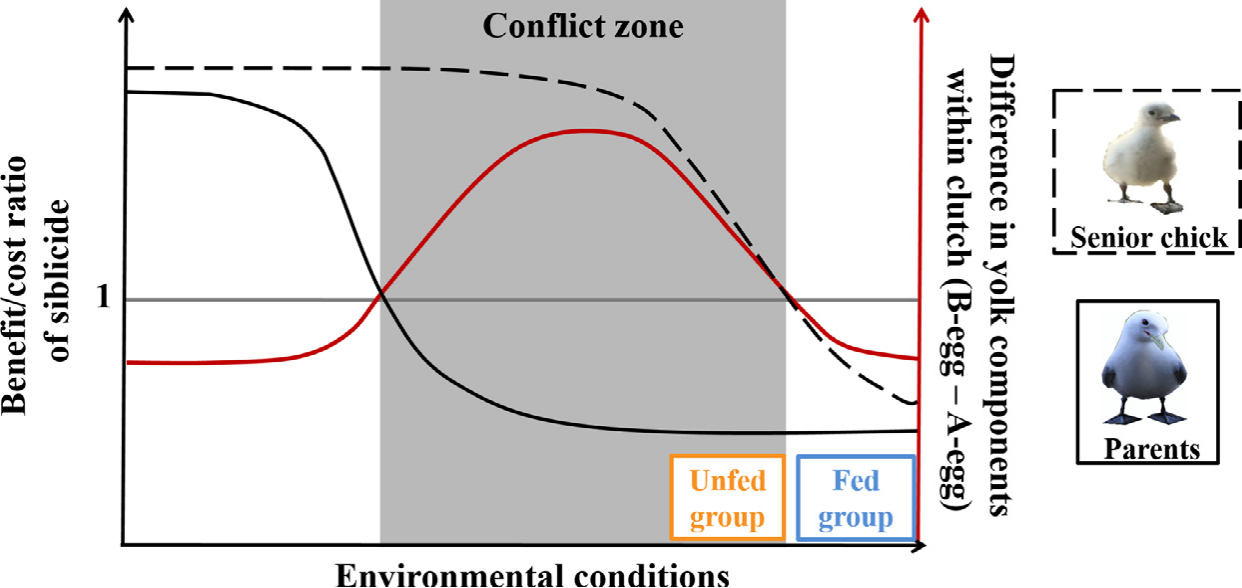
Theoretical representation of (1) the relative benefit (benefit/cost ratio) of siblicide for the senior chick (dashed black line) and the parents (plain black line) (left axis) and (2) the expected within‐clutch difference in yolk testosterone (B‐egg – A‐egg; plain red line) (right axis), according to environmental conditions (*x*‐axis). The shaded area is the conflict zone where siblicide would benefit the senior chick but not the parents, thereby favoring maternal compensation via higher levels of yolk components in the last egg.

Such a bell‐shaped pattern of maternal compensation according to environmental conditions could be explained by a parent–offspring conflict context (Fig. 1). If food is too scarce for the parents to rear all their chicks, the competitive disadvantage of the junior chick(s) might be beneficial from the parents' perspective, because it will facilitate brood reduction (Lack 1947, 1954). For senior chicks, their sibling's death results in more food and thus higher growth rate and fledging prospects. Hence, parents and senior chick(s) agree about the fate of junior chick(s) and we expect maternal compensation to be low in this case. Conversely, when food is plentiful, parents are expected to benefit from junior chick(s) survival. However, maternal compensation may not need to be high as junior chicks' competitive disadvantage is typically low when food availability is high (Drummond 2001). For instance, aggression levels were significantly lower in kittiwake pairs experimentally fed during the chick‐rearing period as compared to control pairs (White et al. 2010). Indeed, for the senior chick, the inclusive fitness benefits/sibling competition cost ratio is probably large in such circumstances. Moreover, previous studies found that maternal effects may also induce costs to chicks in terms of lower hatching success (Navara et al. 2005), longer development (Sockman and Schwabl 2000; Von Engelhardt et al. 2006), immunosuppression (e.g., Groothuis et al. 2005a; Rubolini et al. 2005; Sandell et al. 2009; but see: Tschirren et al. 2005; Müller et al. 2005), increased energy expenditure (Tobler et al. 2007), reduced antioxidant activity (in males only: Tobler and Sandell 2009), nestling survival (Sockman and Schwabl 2000; Muriel et al. 2015), and even maybe long‐term survival (Ruuskanen et al. 2012). Hence, in a situation of high food availability, we expect the benefits of maternal compensation for the junior chick(s) to be outweighed by its costs, thereby reinforcing the selection for low maternal compensation. Yet, we expect the range of environmental conditions leading to these selective pressures to be narrower than in the case of the pressures described for poor environmental conditions, thereby leading to the dissymmetry of the plain red curve reported in Figure 1.

Importantly, parental and senior chick(s) interests are congruent in the extreme situations of exceptionally good or poor environmental conditions. This contrasts with intermediate food availability situations, where a conflict is expected (Fig. 1): Brood reduction might benefit the senior chick(s) because it would increase its share of food, whereas parental fitness might be increased if all chicks survive. We thus expect an increased maternal compensation in these circumstances (e.g., Benowitz‐Fredericks et al. 2013). Facultatively siblicidal species are particularly suitable to test this hypothesis (O'Connor 1978; Mock and Parker 1997) because senior chicks have the faculty to eliminate or accept their younger sibling(s) (Mock et al. 1990).

**Figure 2:**
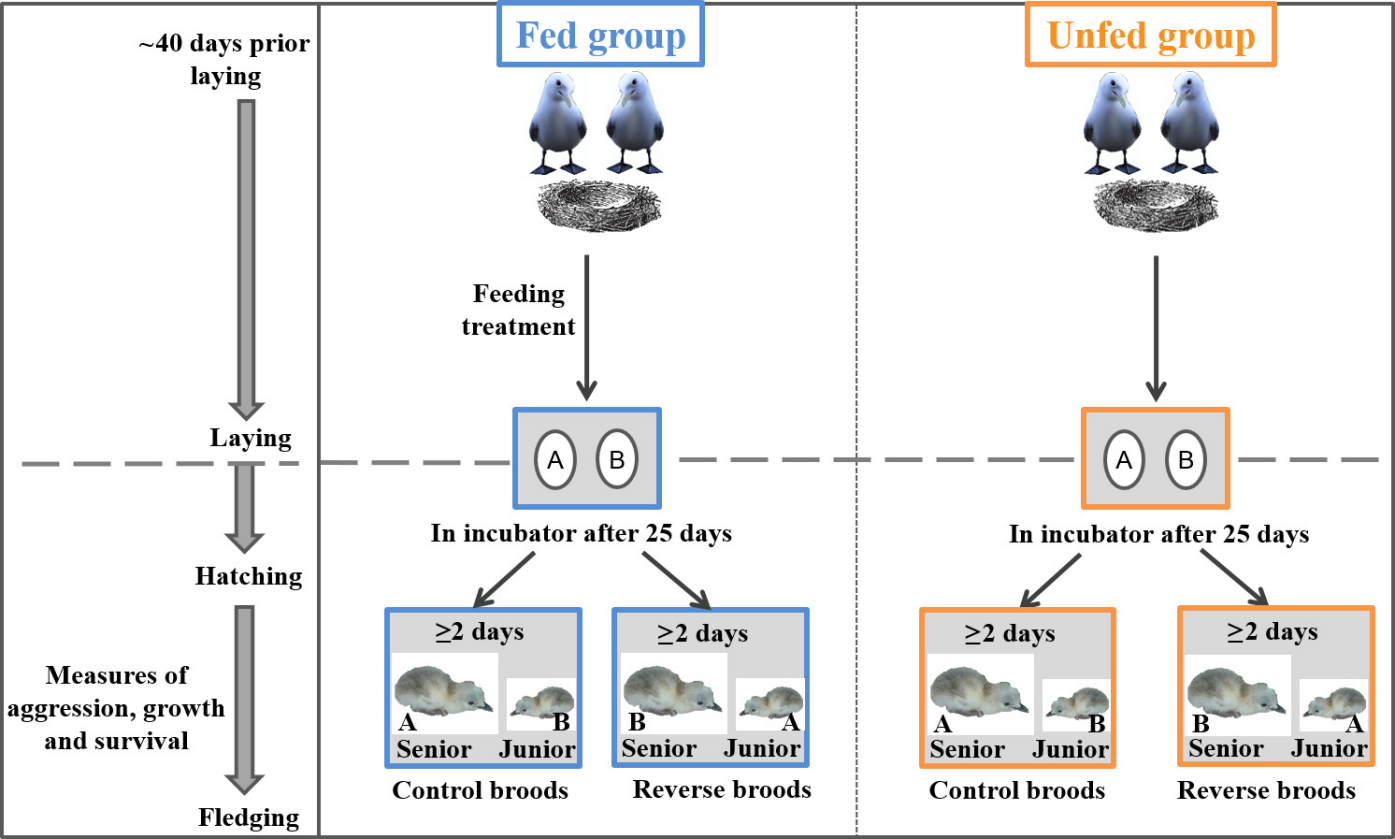
Experimental protocol. Pairs were allocated to the Fed or Unfed groups approximately 40 days before egg laying. Supplemental feeding lasted until the second egg was laid (gray dashed line). Eggs were put in an incubator 25 days after egg laying (i.e., approximately 2 days before hatching). At hatching, two types of experimental broods were created within each feeding treatment: Control broods where the hatching order was maintained (i.e., A‐chick hatching before B‐chick) and Reverse broods where the hatching order was reversed (i.e., B‐chick hatching before A‐chick).

Using the kittiwake, a facultatively siblicidal species (Braun and Hunt 1983; White et al. 2010), we experimentally investigated the consequences for offspring of an environmentally induced variation in maternal effects, as described in Figure 1. We manipulated both prelaying food availability by feeding some breeders (Fed group) but not others (Unfed group) and hatching order (Reverse vs. Control broods) in a 2 × 2 factorial design and recorded chick aggressiveness, growth, and survival (Fig. 2), while statistically controlling for chick sex. By manipulating food availability only before egg laying, we aimed at influencing maternal compensation through yolk components (e.g., Vergauwen et al. 2012), without affecting parental behavior after that stage (Fig. 2). Our experiment was carried out in the same population where the bell‐shaped pattern of yolk testosterone in relation to food availability was found and using the same experimental feeding protocol (Benowitz‐Fredericks et al. 2013). However, the experiment was conducted during a season of relatively higher natural food availability than average (including 2003 and 2004, when occurred the study of Benowitz‐Fredericks et al. 2013): Chick production in 2012 was 0.87 fledglings/nest in control nests, which ranks at the fourth place since 1978 (Hatch 2013) and is higher than chick production in 2003 or 2004 (0.58 and 0.31 fledglings/nest, respectively). Hence, we expected environmental conditions to be on the right side of Figure 1, but with the Fed group obviously representing higher food availability than the Unfed group. Pairs experimentally fed throughout the breeding season (i.e., mimicking exceptionally good conditions) indeed have a consistently higher productivity than control pairs (0.4 more fledglings/nest in average: Vincenzi et al. 2015). We thus hypothesized that the Unfed group was situated on the right of the parent–senior chick conflict zone of Figure 1, whereas the Fed group was situated in the zone of nonconflict on the right of Figure 1. We also suggested that higher yolk hormone allocation occurred in the second egg in the Unfed group as compared to the Fed group, as reported in Benowitz‐Fredericks et al. (2013), although the difference may be lower in our case because of the better environmental conditions. We did not quantify maternal investment in the eggs, because we were interested in chick behavior and yolk biopsies can lead to lower hatching success (e.g., Pilz et al. 2005), thereby potentially decreasing our sample size. We further manipulated hatching order to allow chicks supposed to hatch in a junior position to gain a senior position and thus express more easily the effects of our prelaying feeding treatment on their behavior (full design described in Fig. 2). Indeed, junior chicks typically have a subordinate posture and their behavior is suppressed by senior chicks, leading to very low levels of aggressiveness (Braun and Hunt 1983; Merkling et al. 2014). This manipulation was thus performed to maximize our chances to observe an effect of feeding treatment on our focal behavior.

Our key prediction was that, in Reverse broods, senior chicks (i.e., which hatched from a second‐laid B‐egg) from the Unfed group would be more aggressive, grow, and survive better than senior chicks from the Fed group, whereas prelaying feeding treatment would have no effect on senior chicks in Control broods (i.e., which hatched from a first‐laid A‐egg). In the Unfed group, we also predicted that senior chicks in Reverse broods (i.e., which hatched from a B‐egg) would be more competitive than those in Control broods (i.e., which hatched from an A‐egg). Among junior chicks, we predicted that those of Unfed Control broods (i.e., which hatched from a B‐egg) would be the most competitive, as a result of maternal hormonal deposition in their yolk.

## Materials and Methods

### Study site

The study was carried out from mid‐April to mid‐August 2012 in a population of black‐legged kittiwakes nesting on an abandoned U.S. Air Force radar tower on Middleton Island (59° 26′N, 146° 20′W), Gulf of Alaska. The tower is a 12‐walled polygon where artificial nest sites have been created on the upper walls, permitting observations (from a distance of ~20 cm) through one‐way window glass from inside the tower and allowing us to monitor easily the breeders and their chicks (for more details, see Gill and Hatch 2002).

### Experimental procedure

Upon our arrival, breeding pairs were randomly allocated to the Fed group (*N* = 43) and the Unfed group (*N* = 63; Fig. 2). Fed parents were provided three times daily (08:00, 14:00 and 18:00 h) with capelin *Mallotus villosus* (i.e., a natural prey of kittiwakes, Hatch 2013) until satiation was reached. Food supplementation began on April 20 (41.7 ± 6.3 [mean ± SE] days before laying) and ceased upon laying of the second egg.

Nests were checked twice daily (9:00 and 18:00) throughout the season to record events such as laying, hatching, and chick loss. Laying date was recorded and each egg was individually marked (A for the first‐laid egg and B for the second‐laid egg with nontoxic waterproof ink. Eggs were put into an incubator 25 days after laying (i.e., ~2 days before expected hatching date: Hatch et al. 2009) to monitor hatching closely (Fig. 2). Details of the protocol are given in Merkling et al. (2014).

At hatching, chicks were marked on the head with a nontoxic color marker to identify their hatching rank. We also took blood samples for sexing the chicks (see Merkling et al. 2012 for a detailed protocol) and statistically controlled for that factor. As sexual dimorphism manifests during chick rearing in kittiwakes (Merkling et al. 2012), sex may influence aggressiveness and sibling competition more generally (Uller 2006). Each chick was placed in a foster nest to create two types of experimental broods (“Control broods” and “Reverse broods”, Fig. 2), each containing one A‐chick (born from an A‐egg) and one B‐chick (born from a B‐egg), coming from two different nests. Hence, no parents reared their offspring, and no chicks competed with their sibling. Control broods contained two chicks that hatched slightly more than 2 days apart (51.4 ± 2.82 h, *N *=* *36) which hatching order was maintained (i.e., A‐chick had hatched before B‐chick). Reverse broods also contained two chicks that hatched slightly more than 2 days apart (57.0 ± 1.82 h, *N *=* *28), but which hatching order was reversed (i.e., A‐chick had hatched after B‐chick). Chicks from both types of broods were randomly allocated to adoptive parents of the Fed or Unfed groups, and each brood contained chicks from the same parental treatment.

### Behavioral observations

Chick aggressiveness was estimated using 15 min random focal sampling (Altmann 1974). Each day, the order in which the nests were observed was randomly chosen. Each nest was observed at least once a day, and when time allowed, we randomly picked nests to observe a second or third time. A total of 643 observations (9645 min) were performed on 64 nests. As chick aggression is relatively rare over a 15‐min period (Merkling et al. 2014), we focused on the absence/presence of aggression rather than on the number of aggression during an observation event. Nests were observed from the day the junior chick was placed in the nest until it was 10 days old (i.e., the period when most aggressions occur, White et al. 2010; Leclaire et al. 2011) or one of the chicks died.

### Measuring chick growth

Chicks were measured every 5 days from hatching to 35 days (i.e., close to fledging). We measured head–bill and tarsus length to the nearest 0.1 mm with a caliper, wing length to the nearest 1 mm with a wing ruler, and weight to the nearest 0.1 g using an electronic scale. We estimated growth rate over the first 10 days (i.e., the period of behavioral observations) by taking the scores of the first component of a principal component analysis on wing, tarsus, and head–bill lengths at 0, 5, and 10 days together (96% of total variance explained) and calculating the slope of the linear regression between the scores and age. We also measured the mass gain over the first 10 days with the slope of the linear regression between chick weight and age. We restricted our analyses to individuals coming from nests where both chicks survived at least 10 days (*N = 86*), because we were interested in the effect of our manipulation on chick growth in the context of sibling competition.

To investigate whether our manipulation had lasting effects on chick growth, we also considered maximum measurements (30 or 35 days). To estimate maximum size, we computed a principal component analysis on maximum tarsus, wing, and head–bill length, but as the first principal component explained much less variance (60%) than at earlier ages, we also considered the variables separately to investigate potential trade‐offs between them. We also considered maximum weight. Again, we restricted our analyses to individuals coming from nests where both chicks survived until fledging (*N = 66*).

### Statistical analyses

Following recent recommendations to produce model estimates that are comparable between and within studies (Schielzeth 2010; Grueber et al. 2011), we standardized all input variables by centering and dividing by two standard deviations using the *arm* package (Gelman and Su 2014). We started with a complete model and successively removed terms beginning with those of the highest degree. We tested the change in deviance after removal of a term, using a chi‐square test for mixed models and a *F*‐test for linear models. Whenever an interaction was tested, the main effects comprising the interaction were kept in the model. We separated analyses concerning senior and junior chicks, because considering them together led to model convergence issues for aggression, given the very low number of aggression observed among junior chicks. To be consistent with the analyses of aggression and to facilitate the interpretation of the results, we generalized that approach to all analyses.

Aggressiveness was estimated from GLMM (generalized linear mixed models) with a binomial error distribution and a logit link function, using the *lme4* package (Bates et al. 2011). For senior chicks, the complete model contained the interaction between feeding treatment of the biological parent and hatching rank (A or B), as well as covariates: hatching date, chick age, chick sex, sibling sex, and feeding treatment of the adoptive parents (reported only when significant). In each model, individual identity and biological nest were included as random effects to account for the nonindependence of observations performed on the same individual and of chicks born from the same parents. Observation date and observer identity were also included as random effects. We could not consider the same complete model for junior chicks because the very low number of aggression observed led to extremely large estimates standard errors due to poor model convergence (Bolker et al. 2009). To simplify the model, we omitted the interaction between feeding treatment of the biological parents and hatching rank as well as date and observer random effects.

For all growth variables (growth rate, mass gain and maximum weight, size, wing length, tarsus length, and head–bill length), the complete model for senior and junior chicks contained the same fixed effects as for aggression among senior chicks (see above). We used linear mixed models, with biological nest as a random effect, for the analyses of growth rate and mass gain in senior chicks. For the other variables, as no chicks came from the same biological nest, we used linear models that did not include biological nest as a random factor. For growth of senior chicks, we applied Box–Cox transformation (Box and Cox 1964; using the function provided in the *MASS* package: Venables and Ripley 2002) to meet model assumptions (normality and homoscedasticity of data and residuals).

We used right‐censored data for survival analyses, as hatching date was known for every chick, while survival to fledging was unknown for chicks still alive in the nest when we left the island. A cutoff age of 35 days was applied to fledglings and chicks still alive when we left. We could not test for the interaction between feeding treatment of biological parents and the hatching rank of senior chicks because too few senior chicks died, and the algorithm did not converge. Apart from this limitation, fixed effects of the complete model for senior and junior chicks were the same as for aggression among senior chicks (see above). For senior chicks, we added biological nest as a random effect and used Cox proportional hazards mixed regression models as implemented in the *coxme* package (Therneau 2012). As only two junior chicks came from the same biological nest, we added no random effect for that group.

All analyses were conducted with R 3.0.2 (R Core Team, 2014). Results are shown with mean ± standard error (SE).

## Results

### Chick aggressiveness

**Figure 3:**
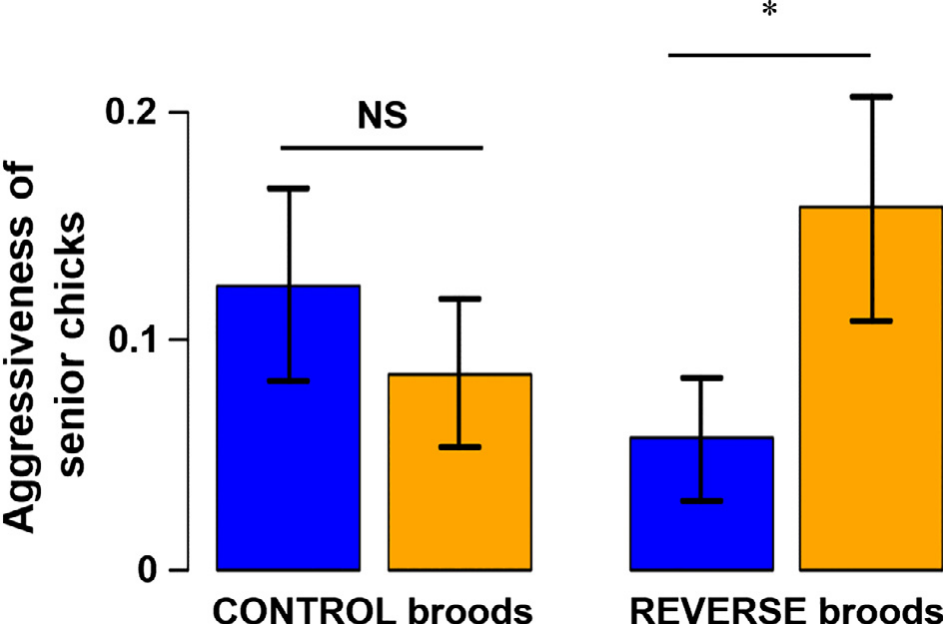
Aggression frequency (±SE) of senior chicks in relation to prelaying feeding treatment of the biological parents and original hatching rank. In Control broods, senior chicks came from A‐eggs, whereas in Reverse broods, they came from B‐eggs. Blue bars: aggression frequencies of chicks born to parents of the Fed group; orange bars: aggression frequency of chicks from parents of the Unfed group. “NS” stands for a nonsignificant difference, whereas “*” is for a significant difference.

As predicted, senior chick aggressiveness showed significant interaction between prelaying feeding treatment of the biological parents and original hatching rank (Table 1). In Reverse broods, senior chicks (from B‐eggs) born to Unfed parents showed higher aggressiveness than senior chicks born to Fed parents (*P* = 0.056), whereas in Control broods (senior chicks from A‐eggs), aggressiveness did not differ between Fed and Unfed groups (*P* = 0.29; Fig. 3). Aggressiveness also decreased significantly with chick age (Table 1).

**Table 1:** Summary of the binomial mixed model describing variation in aggression probability in senior chicks and junior chicks. Significant terms (i.e., retained in the final model) are in bold type. *β* values are the standardized parameter estimates (with their standard errors) taken prior to removal for terms not retained in the final model. *χ²* and *P* are values from the corresponding likelihood‐ratio tests

Parameter	Senior chicks (*N* = 64)			Junior chicks (*N* = 64)		
	*β *± SE	$\chi_{1}^{2}$	*P*	*β *± SE	$\chi_{1}^{2}$	*P*
Intercept	−2.19 ± 0.29			−4.52 ± 0.41		
Biological parent feeding treatment^a^	0.35 ± 0.37		d	0.07 ± 0.83	0.007	0.93
Hatching rank^b^	−0.01 ± 0.33		d	1.03 ± 0.82	3.46	0.063
Chick age	−**0.84 ± 0.31**	**7.96**	**0.005**	−0.73 ± 0.83	0.82	0.36
Chick sex^c^	0.45 ± 0.35	1.64	0.20	−1.20 ± 0.80	2.38	0.12
Foster parent feeding treatment^a^	0.14 ± 0.33	0.18	0.67	1.20 ± 0.91	2.00	0.16
Hatching date	−0.73 ± 0.44	2.59	0.11	−0.11 ± 0.78	0.01	0.91
Sibling sex^c^	0.43 ± 0.36	1.33	0.25	−1.26 ± 0.87	2.73	0.098
Hatching rank^b^ × Biological parent feeding treatment^a^	**1.54 ± 0.66**	**7.25**	**0.007**	–	–	–

–: We did not test for the interaction between hatching rank and the feeding treatment of the biological parents among junior chicks because models did not fit (see Materials and Methods).

a Relative to parents fed before laying.

b Relative to chicks born from an A‐egg.

c Relative to females.

d We did not test for the significance of terms included in significant interactions.

Among junior chicks, however, none of the tested variables were significant on aggressiveness (Table 1). Only hatching rank was close to the significance threshold, with chicks coming from B‐eggs being more aggressive than chicks coming from A‐eggs (Table 1).

### Chick growth

Among senior chicks, mass gain and growth rate were not affected by the feeding treatment of their biological parents, nor by their original hatching rank (Table S1). However, chicks from the Unfed group had significantly smaller maximum wing length than those from the Fed group, whereas this was not the case for maximum weight, size, tarsus length, or head–bill length (Tables S2 and S3).

Among junior chicks, those from the Fed group tended to have a higher growth rate, although not significantly so (*β *± SE: −0.033 ± 0.017; *P* = 0.062), than those from the Unfed group. This relationship, however, disappeared when including an outlier with a very low growth rate (Table S1). Similarly, chicks reared by Unfed adoptive parents grew faster than those reared by Fed adoptive parents (0.036 ± 0.016; *P* = 0.033), but this disappeared when including the same outlier (Table S1). None of the variables of interest influenced mass gain (Table S1) or maximum size, tarsus length, head–bill, or wing length (Table S4).

Among the covariates included in the models, we found that male senior chicks were significantly heavier and reached a larger maximum size than females, the latter result being explained by the longer maximum head–bill length of males (Tables S2 and S3). Moreover, senior chicks with younger male sibling had significantly smaller tarsi and tended to have smaller wings than those with younger female sibling (Table S3). Male junior chicks had a significantly higher maximum weight and reached a larger maximum size than females, the latter result being explained by the longer maximum head–bill and tarsus length of males (Tables S2 and S4). Finally, junior chicks born late in the breeding season reached a smaller maximum size, a result mainly explained by a smaller maximum wing length (Table S4).

### Chick survival

Among senior chicks, neither feeding treatment of the biological parents nor original hatching rank affected chick survival, but we found that males survived better than females (Table S5). None of the other variables were significant (Table S5).

Among junior chicks, no variables significantly affected chick survival. However, chicks from the Unfed group survived less, although not significantly, than those from the Fed group (Table S5).

## Discussion

When food availability is intermediate (i.e., in the Unfed group), parental and senior chick interests are expected to conflict on the fate of junior chicks (Fig. 1). In this context, selection should favor mothers that manipulate their offspring's phenotype by enhancing their junior chick's competitiveness and survival via maternal effects, for instance, by differential allocation of yolk testosterone as suggested by Benowitz‐Fredericks et al. (2013). Here, we experimentally investigated this possibility focusing on chick aggressiveness, growth, and survival using the same population as Benowitz‐Fredericks et al. (2013). As we predicted, senior chicks in Unfed–Reverse broods (hatched from a B‐egg in the Unfed group) were more aggressive that those in Fed–Reverse broods (hatched from a B‐egg in the Fed group), whereas there was no difference in Control broods (among chicks hatched from A‐eggs). This suggests that mothers that were not fed before egg laying compensated for the inferiority of their junior chick by bestowing egg yolks with greater amounts of one or more as yet unidentified components. Contrary to our predictions, however, we could not detect any clear benefits in terms of growth or survival of chicks hatched from B‐eggs in the Unfed group. Our results, however, suggest that chicks hatched from eggs in the Unfed group (regardless of hatching rank) suffered some costs such as reduced growth and survival, although only one effect (i.e., maximum wing length) was significant.

### Egg yolk components

Our results are compatible with the hypothesis that testosterone was the component that laying mothers manipulated in our experiment. It was previously shown in the same population that yolk testosterone levels vary in relation to food availability in a predictable way (Benowitz‐Fredericks et al. 2013): They followed a bell‐shaped pattern with higher levels in B‐eggs when food availability was intermediate. In other populations, kittiwake chicks from eggs injected with androgens are more aggressive than control chicks, although neither begging nor growth or survival increased (Müller et al. 2012). The same research group showed that testosterone‐treated chicks are more aggressive than control chicks, while begging behavior was unchanged (Müller et al. 2014). As we did not measure egg maternal investment, we cannot discard the hypothesis that testosterone was not involved in our results. For instance, corticosterone is another potential candidate as corticosterone‐supplemented kittiwake chicks beg more and are more aggressive than control chicks (Kitaysky et al. 2003). However, although many other yolk components have been shown to influence chick behavior and phenotype (e.g., carotenoid effects on growth: Romano et al. 2008; vitamin E effects on begging: Noguera et al. 2010), testosterone is the principal hormone influencing aggressiveness or dominance and is therefore the most likely candidate underlying our results.

### Chick aggressiveness

As expected, senior chicks in Reverse broods (i.e., hatched from B‐eggs) laid by mothers that were not fed before egg laying (i.e., experiencing parent–senior chick conflict) were more aggressive than all other senior chicks (Fig. 3). However, contrary to our predictions, aggressiveness of junior chicks in Unfed–Control broods (i.e., hatched from a B‐egg) was similar to that of junior chicks born to mothers experimentally fed before egg laying (Fig. 3), although their competitiveness was expected to be favored by their mothers. Only original hatching rank seemed to have an effect with a higher aggressiveness in chicks hatched from B‐eggs. Four studies have already shown that mothers deposit more androgens in B‐ than A‐eggs in kittiwakes (Gasparini et al. 2007; Müller et al. 2012; Vallarino et al. 2012; Benowitz‐Fredericks et al. 2013), which may explain why junior chicks hatched from B‐eggs were more aggressive.

The absence of a prelaying feeding treatment effect on the junior chicks' aggressiveness likely results from the difficulty of measuring intrinsic chick aggressiveness in junior chicks as their behavior seems rapidly inhibited by their older sibling. This raises the question of why would mothers try to increase their junior chick's aggressiveness (e.g., via yolk testosterone) if the chick cannot express it? One reason could be that testosterone increases aggression but also other aspects of competitiveness. For example, direct injection of testosterone into egg yolks of red‐winged blackbirds, *Agelaius phoeniceus*, increased the mass of muscles used for breaking the shell during hatching (Lipar and Ketterson 2000). The same study found that yolk testosterone increased with laying sequence. This strategy might enable mothers to reduce hatching asynchrony by promoting rapid hatching of the last egg. In another study, testosterone increased chick boldness and general activity levels in Japanese quail, *Coturnix japonica* (Niall Daisley et al. 2005). Similarly, kittiwake mothers could favor a suite of competitive abilities in junior chicks by transferring relatively more testosterone into their B‐ than their A‐eggs. This may produce junior chicks that are more “resistant” to their older sibling's attacks by way of counterattack, or better locomotion and alertness facilitating avoidance behavior or simply by increasing their resilience to stress. Further investigations are desirable to understand the influence of testosterone, and maternal effects more generally, on chick behavior in relation to sibling competition (but see Müller and Groothuis 2013).

The fact that 2012 was a season of relatively high food availability (see Introduction) may have led our feeding treatment to have only a small effect on yolk components. This could explain why we did not detect any differences in aggressiveness among junior chicks. Alternatively, our experimental design might also play a role in the absence of differences. Experimental broods were created with hatching asynchrony of approximately 2 days, while the natural asynchrony in this population averages 1.64 days (Merkling et al. 2014). Higher than normal hatching asynchrony may have enhanced the size differential between chicks, reinforced the senior chick's aggressiveness and dominance (Merkling et al. 2014), and increased the subordination of junior chicks relative to hatching asynchrony in unmanipulated broods. Consequently, if mothers in the Unfed group increased the aggressiveness of their junior chicks via maternal effects, any advantage could have been masked by greater asymmetry between chicks.

### Growth and survival

Our results for growth and survival contrast with the findings for aggressiveness, as no interaction between hatching rank and prelaying feeding treatment was detected. In a situation of presumed parent–offspring conflict, mothers seemed to increase the competitiveness of their junior chicks by boosting their aggressiveness but did not seem to accelerate growth. Interestingly, and in line with our results, a previous study on the same species showed that chicks born from androgen‐supplemented eggs were more aggressive, less subordinate, but did not show any growth and survival difference (Müller et al. 2012). If mothers manipulate only chick aggressiveness in a conflict situation, we still would expect higher survival of junior chicks in Unfed–Control broods as compared to those in Unfed–Reverse broods, but that was not the case either. As suggested above, our manipulation of hatching asynchrony likely increased the age difference between chicks, which may have counteracted the advantage given by mothers to their junior chick. Another explanation is that estimating growth and survival is far more demanding in terms of sample size, so that we did not have the statistical power to detect any effect. Furthermore, the high food availability prevailing when we conducted the experiment may also have prevented us from detecting relatively small effects.

Similarly, chick growth and survival were not influenced by original hatching rank either. Rather, we found an effect of prelaying feeding treatment of the biological parents. Specifically, senior chicks born from the Unfed group had shorter wings near fledging than senior chicks from the Fed group, but similar tarsus, head–bill, and mass measurements. Arguably, senior chicks born from the Unfed group faced trade‐offs among different aspects of growth (e.g., Mainwaring et al. 2009). Among junior chicks, the effect of prelaying feeding treatment was less pronounced. Junior chicks born from the Fed group tended to grow faster and survive better than junior chicks born from the Unfed group, further evidence that chicks born from the latter were disadvantaged. We hypothesized that mother in the Fed group were in better condition and laid higher quality eggs that produced higher quality chicks compared to mothers in the Unfed group (e.g., Reynolds et al. 2003; Moreno et al. 2008). This does not necessarily contradict our hypothesis that in intermediate food availability (the Unfed group in our case), mothers benefited by enhancing the competitiveness of their junior chick via maternal effects. Different egg yolk components could be used for different purposes. For instance, in good environmental conditions, mothers may improve the growth and survival of their chicks via higher allocation of yolk substances such as vitamins (Marri and Richner 2014a), antibodies (Abad‐Gómez et al. 2012), and carotenoids (Marri and Richner 2014b).

Males reached a larger maximum size and weight than females among both senior and junior chicks, confirming that sexual dimorphism emerges during the rearing period in this species (Merkling et al. 2012; Vincenzi et al. 2013). As in other species, the sex composition of the brood seemed to influence sibling rivalry (Nathan et al. 2001; Uller 2006; Benhaiem et al. 2012), as senior chicks having a younger male sibling had smaller wings and tarsi than those with a younger female sibling. Owing to their faster growth, sons are more costly to produce and probably require more food than daughters (Merkling et al. 2015). They might thus be more competitive (e.g., Nathan et al. 2001), meaning that senior chicks expend more energy in sibling competition against a younger brother than a younger sister thus hampering growth. While that effect could explain their smaller size, we did not detect any sex difference in aggressiveness, so perhaps another component of sibling competition (e.g., begging) was affected. Surprisingly, we found that female senior chicks were less likely to survive than male senior chicks. This was due apparently to factors other than aggressiveness, because we did not find any sex effect on that variable per se. Curiously, the sex difference in survival did not manifest in junior chicks. Again, it could be that our design imposing slightly greater than normal hatching asynchrony meant that junior chicks of either sex succumbed more readily to the attacks of their senior siblings (Merkling et al. 2014). Lower competitiveness in females may also stem from adaptive differential allocation of maternal resources to male and female eggs or chicks (e.g., Petrie et al. 2001; Rutstein et al. 2005; Badyaev et al. 2006; Abad‐Gómez et al. 2012), but this remains to be investigated in kittiwakes.

Our study illustrates that environmental conditions can influence chick behavior through maternal effects and suggests that mothers tend to adapt the phenotype of their junior chick when food levels are intermediate, that is, when parent–offspring conflict regarding siblicide is expected. Results supported our predictions on aggressiveness in senior chicks, but not on growth and survival. The experiment emphasizes the condition‐dependence of maternal effects. More information about the benefit/cost ratio of siblicide, and brood reduction more generally, will help to understand when maternal effects should be used by mothers to influence their chicks' survival. Moreover, as it seems that different yolk components have different effects on chick phenotypes, it would be of interest to study whether mothers adaptively adjust their relative levels at the egg stage.

## Conflict of Interest

None declared.

## Supporting information


**Table S1.** Summary of the linear mixed models and linear models describing variation in growth rate and mass gain in the first 10 days in senior chicks (*N* = 39) and junior chicks (*N* = 39, outlier included), respectively.
**Table S2.** Summary of the linear models describing variation in maximum weight in senior chicks (*N* = 33) and junior chicks (*N* = 33, outlier included), respectively.
**Table S3.** Summary of the linear models describing variation in maximum size (PCA score), tarsus, head‐bill and wing lengths in senior chicks (*N* = 33).
**Table S4.** Summary of the linear models describing variation in maximum size (PCA score), tarsus, head‐bill and wing lengths in junior chicks (*N* = 33).
**Table S5.** Summary of the linear models describing variation in survival in senior chicks (*N* = 64) and junior chicks (*N* = 64), respectively.

## References

[ece31777-bib-0001] Abad‐Gómez, J. M. , J. A. Masero , A. Villegas , N. Albano , J. S. Gutiérrez , and J. M. Sánchez‐Guzmán . 2012 Sex‐specific deposition and survival effects of maternal antibodies: a case study with the gull‐billed tern *Gelochelidon nilotica* . J. Avian Biol. 43:491–495.

[ece31777-bib-0002] Altmann, J. 1974 Observational study of behavior: sampling methods. Behaviour 49:3–4. 10.1163/156853974x00534 4597405

[ece31777-bib-0003] Badyaev, A. V. , D. Acevedo Seaman , K. J. Navara , G. E. Hill , and M. T. Mendonca . 2006 Evolution of sex‐biased maternal effects in birds: III. Adjustment of ovulation order can enable sex‐specific allocation of hormones, carotenoids, and vitamins. J. Evol. Biol. 19:1044–1057. 16780506 10.1111/j.1420-9101.2006.01106.x

[ece31777-bib-0004] Bates, D. , M. Maechler , and B. M. Bolker . 2011 Package “lme4”: linear mixed‐effects models using S4 classes (version 0.999375‐42).

[ece31777-bib-0005] Benhaiem, S. , H. Hofer , S. Kramer‐Schadt , E. Brunner , and M. L. East . 2012 Sibling rivalry: training effects, emergence of dominance and incomplete control. Proc. R. Soc. Lond. B Biol. Sci. 279:3727–3735. 10.1098/rspb.2012.0925 PMC3415906 22719032

[ece31777-bib-0006] Benowitz‐Fredericks, Z. M. , A. S. Kitaysky , J. Welcker , and S. A. Hatch . 2013 Effects of food availability on yolk androgen deposition in the black‐legged kittiwake (*Rissa tridactyla*), a seabird with facultative brood reduction. PLoS ONE 8:e62949. 23675443 10.1371/journal.pone.0062949 PMC3652864

[ece31777-bib-0007] Bolker, B. M. , M. E. Brooks , C. J. Clark , S. W. Geange , J. R. Poulsen , M. H. H. Stevens , et al. 2009 Generalized linear mixed models: a practical guide for ecology and evolution. Trends Ecol. Evol. 24:127–135. 19185386 10.1016/j.tree.2008.10.008

[ece31777-bib-0008] Box, G. , and D. Cox . 1964 An analysis of transformations. J. R. Stat. Soc. B. Methodol. 26:211–252.

[ece31777-bib-0009] Braun, B. M. , and G. L. J. Hunt . 1983 Brood reduction in black‐legged kittiwakes. Auk 100:469–476.

[ece31777-bib-0010] De Fraipont, M. , J. Clobert , H. John‐Alder , and S. Meylan . 2000 Increased pre‐natal maternal corticosterone promotes philopatry of offspring in common lizards *Lacerta vivipara* . J. Anim. Ecol. 69:404–413.

[ece31777-bib-0011] Drummond, H. 2001 A revaluation of the role of food in broodmate aggression. Anim. Behav. 61:517–526.

[ece31777-bib-0012] Eising, C. M. , C. Eikenaar , H. Schwabl , and T. G. G. Groothuis . 2001 Maternal androgens in black‐headed gull (*Larus ridibundus*) eggs: consequences for chick development. Proc. R. Soc. Lond. B Biol. Sci. 268:839–846. 10.1098/rspb.2001.1594 PMC1088678 11345330

[ece31777-bib-0013] Gasparini, J. , T. Boulinier , V. A. Gill , D. Gil , S. A. Hatch , and A. Roulin . 2007 Food availability affects the maternal transfer of androgens and antibodies into eggs of a colonial seabird. J. Evol. Biol. 20:874–880. 17465898 10.1111/j.1420-9101.2007.01315.x

[ece31777-bib-0014] Gelman, A. , and Y.‐S. Su . 2014 arm: data analysis using regression and multilevel/hierarchical models. . R package version 1.7‐03.

[ece31777-bib-0015] Gill, V. A. , and S. A. Hatch . 2002 Components of productivity in black‐legged kittiwakes *Rissa tridactyla*: response to supplemental feeding. J. Avian Biol. 33:113–126.

[ece31777-bib-0016] Groothuis, T. G. G. , and H. Schwabl . 2008 Hormone‐mediated maternal effects in birds: mechanisms matter but what do we know of them?. Philos. Trans. R. Soc. Lond. B Biol. Sci. 363:1647–1661. 18048291 10.1098/rstb.2007.0007 PMC2606725

[ece31777-bib-0017] Groothuis, T. G. G. , C. M. Eising , C. Dijkstra , and W. Müller . 2005a Balancing between costs and benefits of maternal hormone deposition in avian eggs. Biol. Lett. 1:78–81. 17148133 10.1098/rsbl.2004.0233 PMC1629043

[ece31777-bib-0018] Groothuis, T. G. G. , W. Müller , N. von Engelhardt , C. Carere , and C. Eising . 2005b Maternal hormones as a tool to adjust offspring phenotype in avian species. Neurosci. Biobehav. Rev. 29:329–352. 15811503 10.1016/j.neubiorev.2004.12.002

[ece31777-bib-0019] Grueber, C. , S. Nakagawa , R. Laws , and I. Jamieson . 2011 Multimodel inference in ecology and evolution: challenges and solutions. J. Evol. Biol. 24:699–711. 21272107 10.1111/j.1420-9101.2010.02210.x

[ece31777-bib-0020] Gutterman, Y. 2000 Maternal effects on seeds during development Pp. 59–84 *in* Fenner M., ed. Seed: the ecology of regeneration in plant communities, 2nd edn CABI Publishing, Oxon, U.K.

[ece31777-bib-0021] Hasselquist, D. , and J.‐A. Nilsson . 2009 Maternal transfer of antibodies in vertebrates: trans‐generational effects on offspring immunity. Philos. Trans. R. Soc. Lond. B Biol. Sci. 364:51–60. 18926976 10.1098/rstb.2008.0137 PMC2666691

[ece31777-bib-0022] Hatch, S. A. 2013 Kittiwake diets and chick production signal a 2008 regime shift in the Northeast Pacific. Mar. Ecol. Prog. Ser. 477:271–284.

[ece31777-bib-0023] Hatch, S. A. , G. J. Robertson , and H. P. Baird . 2009 Black‐legged kittiwake (Rissa tridactyla). The birds of North America online. Cornell Laboratory of Ornithology, Ithaca, NY.

[ece31777-bib-0024] Kitaysky, A. S. , E. Kitaiskaia , J. Piatt , and J. C. Wingfield . 2003 Benefits and costs of increased levels of corticosterone in seabird chicks. Horm. Behav. 43:140–149. 12614644 10.1016/s0018-506x(02)00030-2

[ece31777-bib-0025] Lack, D. 1947 The significance of clutch‐size. Ibis 89:302–352.

[ece31777-bib-0026] Lack, D. 1954 The natural regulation of animal numbers. Clarendon Press, Oxford, U.K.

[ece31777-bib-0027] Leclaire, S. , V. Bourret , R. H. Wagner , S. A. Hatch , F. Helfenstein , O. Chastel , et al. 2011 Behavioral and physiological responses to male handicap in chick‐rearing black‐legged kittiwakes. Behav. Ecol. 22:1156–1165.

[ece31777-bib-0028] Lipar, J. L. , and E. D. Ketterson . 2000 Maternally derived yolk testosterone enhances the development of the hatching muscle in the red‐winged blackbird *Agelaius phoeniceus* . Proc. R. Soc. Lond. B Biol. Sci. 267:2005–2010. 10.1098/rspb.2000.1242 PMC1690769 11075714

[ece31777-bib-0029] Maestripieri, D. , and J. M. Mateo . 2009 Maternal effects in mammals. University of Chicago Press, Chicago, IL.

[ece31777-bib-0030] Mainwaring, M. C. , L. V. Rowe , D. J. Kelly , J. Grey , S. Bearhop , and I. R. Hartley . 2009 Hatching asynchrony and growth trade‐offs within barn swallow broods. Condor 111:668–674.

[ece31777-bib-0031] Marri, V. , and H. Richner . 2014a Differential effects of vitamins E and C and carotenoids on growth, resistance to oxidative stress, fledging success and plumage colouration in wild great tits. J. Exp. Biol. 217:1478–1484. 24436384 10.1242/jeb.096826

[ece31777-bib-0032] Marri, V. , and H. Richner . 2014b Yolk carotenoids increase fledging success in great tit nestlings. Oecologia 176:371–377. 25142046 10.1007/s00442-014-3051-2

[ece31777-bib-0033] Marshall, D. J. , and T. Uller . 2007 When is a maternal effect adaptive? Oikos 116:1957–1963.

[ece31777-bib-0034] Merkling, T. , S. Leclaire , E. Danchin , E. Lhuillier , R. H. Wagner , J. White , et al. 2012 Food availability and offspring sex in a monogamous seabird: insights from an experimental approach. Behav. Ecol. 23:751–758.

[ece31777-bib-0035] Merkling, T. , L. Agdere , E. Albert , R. Durieux , S. A. Hatch , E. Danchin , et al. 2014 Is natural hatching asynchrony optimal? An experimental investigation of sibling competition patterns in a facultatively siblicidal seabird. Behav. Ecol. Sociobiol. 68:309–319.

[ece31777-bib-0036] Merkling, T. , J. Welcker , A. J. M. Hewison , S. A. Hatch , A. S. Kitaysky , J. R. Speakman , et al. 2015 Identifying the selective pressures underlying offspring sex‐ratio adjustments: a case study in a wild seabird. Behav. Ecol. 26:916–925.

[ece31777-bib-0037] Mock, D. W. , and G. A. Parker . 1997 The evolution of sibling rivalry. Oxford Univ. Press, Oxford, U.K.

[ece31777-bib-0038] Mock, D. W. , H. Drummond , and C. H. Stinson . 1990 Avian siblicide. Am. Sci. 78:438–449.

[ece31777-bib-0039] Moreno, J. , E. Lobato , J. Morales , S. Merino , J. Martínez‐De La Puente , and G. Tomás . 2008 Pre‐laying nutrition mediates maternal effects on offspring immune capacity and growth in the pied flycatcher. Oecologia 156:727–735. 18369666 10.1007/s00442-008-1029-7

[ece31777-bib-0040] Mousseau, T. A. , and H. Dingle . 1991 Maternal effects in insect life histories. Annu. Rev. Entomol. 36:511–534.

[ece31777-bib-0041] Mousseau, T. A. , and C. W. Fox . 1998 The adaptive significance of maternal effects. Trends Ecol. Evol. 13:403–407. 21238360 10.1016/s0169-5347(98)01472-4

[ece31777-bib-0042] Müller, M. S. , and T. G. G. Groothuis . 2013 Within‐clutch variation in yolk testosterone as an adaptive maternal effect to modulate avian sibling competition: evidence from a comparative study. Am. Nat. 181:125–136. 23234850 10.1086/668601

[ece31777-bib-0043] Müller, W. , T. G. G. Groothuis , A. Kasprzik , C. Dijkstra , R. V. Alatalo , and H. Siitari . 2005 Prenatal androgen exposure modulates cellular and humoral immune function of black‐headed gull chicks. Proc. R. Soc. Lond. B Biol. Sci. 272:1971–1977. 10.1098/rspb.2005.3178 PMC1559883 16191605

[ece31777-bib-0044] Müller, W. , P. Korsten , and N. von Engelhardt . 2007 Manipulative signals in family conflict? On the function of maternal yolk hormones in birds. Am. Nat. 169:E84–E96. 17253431 10.1086/511962

[ece31777-bib-0045] Müller, M. S. , Y. Roelofs , K. E. Erikstad , and T. G. G. Groothuis . 2012 Maternal androgens increase sibling aggression, dominance, and competitive ability in the siblicidal black‐legged kittiwake (*Rissa tridactyla*). PLoS ONE 7:e47763. 23112843 10.1371/journal.pone.0047763 PMC3480423

[ece31777-bib-0046] Müller, M. S. , B. Moe , and T. G. G. Groothuis . 2014 Testosterone increases siblicidal aggression in black‐legged kittiwake chicks (*Rissa tridactyla*). Behav. Ecol. Sociobiol. 68:223–232.

[ece31777-bib-0047] Muriel, J. , P. Salmón , A. Nunez‐Buiza , F. Salas , L. Pérez‐Rodríguez , M. Puerta , et al. 2015 Context‐dependent effects of yolk androgens on nestling growth and immune function in a multibrooded passerine. J. Evol. Biol. 28:1476–1488. 26079258 10.1111/jeb.12668

[ece31777-bib-0048] Nathan, A. , S. Legge , and A. Cockburn . 2001 Nestling aggression in broods of a siblicidal kingfisher, the laughing kookaburra. Behav. Ecol. 12:716–725.

[ece31777-bib-0049] Navara, K. J. , G. E. Hill , and M. T. Mendonça . 2005 Variable effects of yolk androgens on growth, survival, and immunity in eastern bluebird nestlings. Physiol. Biochem. Zool. 78:570–578. 15957111 10.1086/430689

[ece31777-bib-0050] Niall Daisley, J. , V. Bromundt , E. Möstl , and K. Kotrschal . 2005 Enhanced yolk testosterone influences behavioral phenotype independent of sex in Japanese quail chicks *Coturnix japonica* . Horm. Behav. 47:185–194. 15664022 10.1016/j.yhbeh.2004.09.006

[ece31777-bib-0051] Noguera, J. C. , J. Morales , C. Pérez , and A. Velando . 2010 On the oxidative cost of begging: antioxidants enhance vocalizations in gull chicks. Behav. Ecol. 21:479–484.

[ece31777-bib-0052] O'Connor, R. J. 1978 Brood reduction in birds: selection for fratricide, infanticide and suicide? Anim. Behav. 26:79–96.

[ece31777-bib-0053] Petrie, M. , H. Schwabl , N. Brande‐Lavridsen , and T. Burke . 2001 Maternal investment – sex differences in avian yolk hormone levels. Nature 412:498. 11484039 10.1038/35087652

[ece31777-bib-0054] Pilz, K. M. , E. Adkins‐Regan , and H. Schwabl . 2005 No sex difference in yolk steroid concentrations of avian eggs at laying. Biol. Lett. 1:318–321. 17148197 10.1098/rsbl.2005.0321 PMC1617136

[ece31777-bib-0055] R Core Team . 2014 R: a language and environment for statistical computing. R Foundation for Statistical Computing, Vienna, Austria.

[ece31777-bib-0056] Reynolds, S. J. , S. J. Schoech , and R. Bowman . 2003 Diet quality during pre‐laying and nestling periods influences growth and survival of Florida scrub‐jay (*Aphelocoma coerulescens*) chicks. J. Zool. 261:217–226.

[ece31777-bib-0057] Romano, M. , M. Caprioli , R. Ambrosini , D. Rubolini , M. Fasola , and N. Saino . 2008 Maternal allocation strategies and differential effects of yolk carotenoids on the phenotype and viability of yellow‐legged gull (*Larus michahellis*) chicks in relation to sex and laying order. J. Evol. Biol. 21:1626–1640. 18713240 10.1111/j.1420-9101.2008.01599.x

[ece31777-bib-0058] Rubolini, D. , M. Romano , G. Boncoraglio , R. P. Ferrari , R. Martinelli , P. Galeotti , et al. 2005 Effects of elevated egg corticosterone levels on behavior, growth, and immunity of yellow‐legged gull (*Larus michahellis*) chicks. Horm. Behav. 47:592–605. 15811362 10.1016/j.yhbeh.2005.01.006

[ece31777-bib-0059] Rutstein, A. N. , L. Gilbert , P. J. Slater , and J. A. Graves . 2005 Sex‐specific patterns of yolk androgen allocation depend on maternal diet in the zebra finch. Behav. Ecol. 16:62–69.

[ece31777-bib-0060] Ruuskanen, S. , B. Doligez , L. Gustafsson , and T. Laaksonen . 2012 Long‐term effects of yolk androgens on phenotype and parental feeding behavior in a wild passerine. Behav. Ecol. Sociobiol. 66:1201–1211.

[ece31777-bib-0061] Saino, N. , R. Ferrari , M. Romano , R. Martinelli , and A. P. Møller . 2003 Experimental manipulation of egg carotenoids affects immunity of barn swallow nestlings. Proc. R. Soc. Lond. B Biol. Sci. 270:2485–2489. 10.1098/rspb.2003.2534 PMC1691538 14667340

[ece31777-bib-0062] Sandell, M. I. , M. Tobler , and D. Hasselquist . 2009 Yolk androgens and the development of avian immunity: an experiment in jackdaws (*Corvus monedula*). J. Exp. Biol. 212:815–822. 19251998 10.1242/jeb.022111

[ece31777-bib-0063] Schielzeth, H. 2010 Simple means to improve the interpretability of regression coefficients. Methods Ecol. Evol. 1:103–113.

[ece31777-bib-0064] Sockman, K. W. , and H. Schwabl . 2000 Yolk androgens reduce offspring survival. Proc. R. Soc. Lond. B Biol. Sci. 267:1451–1456. 10.1098/rspb.2000.1163 PMC1690699 10983830

[ece31777-bib-0065] Therneau, T. 2012 Package “coxme”: mixed effects Cox models (version 2.2‐3).

[ece31777-bib-0066] Tobler, M. , and M. I. Sandell . 2009 Sex‐specific effects of prenatal testosterone on nestling plasma antioxidant capacity in the zebra finch. J. Exp. Biol. 212:89–94. 19088214 10.1242/jeb.020826

[ece31777-bib-0067] Tobler, M. , J. A. Nilsson , and J. F. Nilsson . 2007 Costly steroids: egg testosterone modulates nestling metabolic rate in the zebra finch. Biol. Lett. 3:408–410. 17456447 10.1098/rsbl.2007.0127 PMC2390662

[ece31777-bib-0068] Tschirren, B. , V. Saladin , P. S. Fitze , H. Schwabl , and H. Richner . 2005 Maternal yolk testosterone does not modulate parasite susceptibility or immune function in great tit nestlings. J. Anim. Ecol. 74:675–682.

[ece31777-bib-0069] Uller, T. 2006 Sex‐specific sibling interactions and offspring fitness in vertebrates: patterns and implications for maternal sex ratios. Biol. Rev. 81:207–217. 16677432 10.1017/S1464793105006962

[ece31777-bib-0070] Uller, T. 2008 Developmental plasticity and the evolution of parental effects. Trends Ecol. Evol. 23:432–438. 18586350 10.1016/j.tree.2008.04.005

[ece31777-bib-0071] Vallarino, A. , N. Evans , F. Daunt , S. Wanless , and R. Nager . 2012 Egg components vary independently of each other in the facultative siblicidal Black‐legged Kittiwake *Rissa tridactyla* . J. Ornithol. 153:513–523.

[ece31777-bib-0072] Venables, W. N. , and B. D. Ripley . 2002 Modern applied statistics with S. Springer, New York, NY.

[ece31777-bib-0073] Verboven, N. , P. Monaghan , D. M. Evans , H. Schwabl , N. Evans , C. Whitelaw , et al. 2003 Maternal condition, yolk androgens and offspring performance: a supplemental feeding experiment in the lesser black‐backed gull (*Larus fuscus*). Proc. R. Soc. Lond. B Biol. Sci. 270:2223–2232. 10.1098/rspb.2003.2496 PMC1691499 14613608

[ece31777-bib-0074] Vergauwen, J. , V. C. Goerlich , T. G. G. Groothuis , M. Eens , and W. Müller . 2012 Food conditions affect yolk testosterone deposition but not incubation attendance. Gen. Comp. Endocrinol. 176:112–119. 22265816 10.1016/j.ygcen.2012.01.003

[ece31777-bib-0075] Vincenzi, S. , S. A. Hatch , M. Mangel , and A. S. Kitaysky . 2013 Food availability affects onset of reproduction in a long‐lived seabird. Proc. R. Soc. Lond. B Biol. Sci. 280:20130554. 10.1098/rspb.2013.0554 PMC3652464 23576791

[ece31777-bib-0076] Vincenzi, S. , S. A. Hatch , T. Merkling , and A. S. Kitaysky . 2015 Carry‐over effects of food supplementation on recruitment and breeding performance of long‐lived seabirds. Proc. R. Soc. Lond. B Biol. Sci. 282:20150762. 10.1098/rspb.2015.0762 PMC4528510 26180065

[ece31777-bib-0077] Von Engelhardt, N. , C. Carere , C. Dijkstra , and T. G. G. Groothuis . 2006 Sex‐specific effects of yolk testosterone on survival, begging and growth of zebra finches. Proc. R. Soc. Lond. B Biol. Sci. 273:65–70. 10.1098/rspb.2005.3274 PMC1560008 16519236

[ece31777-bib-0078] White, J. , S. Leclaire , M. Kriloff , H. Mulard , S. A. Hatch , and E. Danchin . 2010 Sustained increase in food supplies reduces broodmate aggression in black‐legged kittiwakes. Anim. Behav. 79:1095–1100.

[ece31777-bib-0079] Wolf, J. B. , and M. J. Wade . 2009 What are maternal effects (and what are they not)? Philos. Trans. R. Soc. Lond. B Biol. Sci. 364:1107–1115. 19324615 10.1098/rstb.2008.0238 PMC2666680

