# Supplementary material for: Maternal effects as drivers of sibling competition in a parent–offspring conflict context? An experimental test
Source: Ecol Evol. 2016 May 3;6(11):3699–710. doi: 10.1002/ece3.1777 (PMC5513303; doi:10.1002/ece3.1777)
Supplement: Supplementary file 1 — Table S1. Summary of the linear mixed models and linear models describing variation in growth rate and mass gain in the first 10 days in senior chicks (N = 39) and junior chicks (N = 39, outlier included), respectively. Table S2. Summary of the linear models describing variation in maximum weight in senior chicks (N = 33) and junior chicks (N = 33, outlier included), respectively. Table S3. Summary of the linear models describing variation in maximum size (PCA score), tarsus, head‐bill and wing lengths in senior chicks (N = 33). Table S4. Summary of the linear models describing variation in maximum size (PCA score), tarsus, head‐bill and wing lengths in junior chicks (N = 33). Table S5. Summary of the linear models describing variation in survival in senior chicks (N = 64) and junior chicks (N = 64), respectively. [file ECE3-6-3699-s001.docx]

**Table S1.** Summary of the linear mixed models and linear models describing variation in growth rate and mass gain in the first 10 days in senior chicks (N = 39) and junior chicks (N = 39, outlier included), respectively. Significant terms (i.e. retained in the final model) are in bold type. β are the standardized parameter estimates (with their standard errors) taken prior to removal for terms not retained in the final model.

|  | Senior chicks: mass gain | | | Senior chicks: growth rate | | | Junior chicks: mass gain | | | Junior chicks: growth rate | | |
| --- | --- | --- | --- | --- | --- | --- | --- | --- | --- | --- | --- | --- |
| Parameter | β ± SE | χ²_1_ | *P* | β ± SE | χ²_1_ | *P* | β ± SE | F_32,33_ | *P* | β ± SE | F_32,33_ | *P* |
| Intercept | 14.26 ± 0.30 |  |  | -0.27 ± 0.001 |  |  | 13.09 ± 0.45 |  |  | 0.37 ± 0.01 |  |  |
| Biologic parent feeding treatment^a^ | 0.38 ± 0.66 | 0.32 | 0.57 | 0.001 ± 0.002 | 0.47 | 0.55 | 0.04 ± 0.99 | 0.01 | 0.97 | -0.025 ± 0.023 | 1.20 | 0.28 |
| Hatching rank^b^ | 0.92 ± 0.64 | 1.94 | 0.16 | 0.001 ± 0.002 | 0.05 | 0.83 | -0.43 ± 0.97 | 0.20 | 0.65 | -0.003 ± 0.023 | 0.02 | 0.88 |
| Chick sex^c^ | 0.87 ± 0.62 | 1.88 | 0.17 | 0.002 ± 0.002 | 0.88 | 0.35 | -0.74 ± 0.96 | 0.49 | 0.55 | -0.024 ± 0.023 | 1.15 | 0.29 |
| Foster parent feeding treatment^a^ | 0.66 ± 0.62 | 1.13 | 0.29 | 0.001 ± 0.002 | 0.22 | 0.64 | 0.57 ± 0.92 | 0.38 | 0.54 | 0.019 ± 0.022 | 0.78 | 0.38 |
| Hatching date | -0.39 ± 0.67 | 0.34 | 0.56 | -0.003 ± 0.002 | 1.83 | 0.18 | -0.37 ± 0.99 | 0.14 | 0.71 | -0.015 ± 0.024 | 0.43 | 0.51 |
| Sibling sex^c^ | 0.78 ± 0.64 | 1.43 | 0.23 | 0.002 ± 0.002 | 0.73 | 0.39 | -0.73 ± 0.92 | 0.62 | 0.44 | -0.016 ± 0.022 | 0.43 | 0.57 |
| Biologic parent feeding treatment^a^ × Hatching rank^b^ | -1.36 ± 1.21 | 1.13 | 0.28 | -0.001 ± 0.004 | 0.13 | 0.72 | 3.16 ± 1.85 | 2.92 | 0.09 | 0.055 ± 0.045 | 1.50 | 0.23 |

^a^ Relative to parents Fed before laying

^b^ Relative to chicks born from an A-egg

^c^ Relative to females

**Table S2.** Summary of the linear models describing variation in maximum weight in senior chicks (N = 33) and junior chicks (N = 33, outlier included), respectively. Significant terms (i.e. retained in the final model) are in bold type. β are the standardized parameter estimates (with their standard errors) taken prior to removal for terms not retained in the final model.

|  | Senior chicks | | | Junior chicks | | |
| --- | --- | --- | --- | --- | --- | --- |
| Parameter | β ± SE | F_26,27_ | P | β ± SE | F_26,27_ | P |
| Intercept | 426.136 ± 4.77 |  |  | 428.00 ± 4.35 |  |  |
| Biologic parent feeding treatment^a^ | 16.64 ± 9.84 | 2.86 | 0.10 | 12.73 ± 9.14 | 1.94 | 0.17 |
| Hatching rank^b^ | -4.22 ± 10.06 | 0.18 | 0.68 | 9.60 ± 9.45 | 1.03 | 0.32 |
| Chick sex^c^ | **27.84 ± 9.76** | **8.81** | **0.006** | **30.59 ± 8.92** | **12.12** | **0.002** |
| Foster parent feeding treatment^a^ | 16.95 ± 9.78 | 3.00 | 0.095 | -10.13 ± 9.05 | 1.25 | 0.27 |
| Hatching date | 5.32 ± 10.47 | 0.26 | 0.61 | 4.12 ± 9.78 | 0.18 | 0.68 |
| Sibling sex^c^ | 5.28 ± 9.80 | 0.29 | 0.59 | -12.15 ± 8.94 | 1.84 | 0.19 |
| Biologic parent feeding treatment^a^ × Hatching rank^b^ | 5.28 ± 20.0 | 0.07 | 0.79 | -15.24 ± 18.49 | 0.68 | 0.42 |

^a^ Relative to parents Fed before laying

^b^ Relative to chicks born from an A-egg

^c^ Relative to females

**Table S3.** Summary of the linear models describing variation in maximum size (PCA score), tarsus, head-bill and wing lengths in senior chicks (N = 33). Significant terms (i.e. retained in the final model) are in bold type. β are the standardized parameter estimates (with their standard errors) taken prior to removal for terms not retained in the final model.

|  | Maximum size | | | Maximum tarsus length | | | Maximum head-bill length | | | Maximum wing length | | |
| --- | --- | --- | --- | --- | --- | --- | --- | --- | --- | --- | --- | --- |
| Parameter | β ± SE | F_26,27_ | *P* | β ± SE | F_26,27_ | *P* | β ± SE | F_26,27_ | *P* | β ± SE | F_26,27_ | *P* |
| Intercept | 0.29 ± 0.21 |  |  | 36.33 ± 0.20 |  |  | 85.32 ± 0.40 |  |  | 249.55 ± 1.83 |  |  |
| Biologic parent feeding treatment^a^ | -0.63 ± 0.44 | 2.03 | 0.16 | -0.63 ± 0.41 | 2.40 | 0.13 | 0.56 ± 0.83 | 0.46 | 0.50 | **-9.31 ± 3.65** | **10.06** | **0.004** |
| Hatching rank^b^ | -0.39 ± 0.45 | 0.74 | 0.40 | -0.51 ±0.41 | 1.53 | 0.23 | 0.50 ± 0.85 | 0.34 | 0.56 | -6.61 ± 3.73 | 3.14 | 0.08 |
| Chick sex^c^ | **0.93 ± 0.42** | **4.27** | **0.05** | 0.58 ± 0.39 | 2.14 | 0.16 | **2.18 ± 0.82** | **7.73** | **0.01** | 2.08 ± 3.55 | 0.34 | 0.56 |
| Foster parent feeding treatment^a^ | -0.33 ± 0.44 | 0.57 | 0.46 | -0.27 ± 0.40 | 0.45 | 0.51 | 0.18 ± 0.82 | 0.05 | 0.83 | -6.03 ± 3.62 | 2.77 | 0.11 |
| Hatching date | 0.63 ± 0.47 | 1.81 | 0.19 | 0.03 ± 0.43 | 0.01 | 0.94 | 1.59 ± 0.88 | 3.24 | 0.08 | 5.72 ± 3.89 | 2.17 | 0.15 |
| Sibling sex^c^ | -0.50 ± 0.44 | 1.28 | 0.27 | **-0.66 ± 0.41** | **4.45** | **0.045** | 0.74 ± 0.83 | 0.80 | 0.38 | -6.92 ± 3.63 | 3.63 | 0.068 |
| Biologic parent feeding treatment^a^ × Hatching rank^b^ | -1.11 ± 0.87 | 1.63 | 0.21 | -0.33 ± 0.83 | 0.16 | 0.69 | -1.99 ± 1.65 | 1.46 | 0.24 | -11.80 ± 7.06 | 2.80 | 0.11 |

^a^ Relative to parents Fed before laying

^b^ Relative to chicks born from an A-egg

^c^ Relative to females

**Table S4.** Summary of the linear models describing variation in maximum size (PCA score), tarsus, head-bill and wing lengths in junior chicks (N = 33). Significant terms (i.e. retained in the final model) are in bold type. β are the standardized parameter estimates (with their standard errors) taken prior to removal for terms not retained in the final model.

|  | Maximum size | | | Maximum tarsus length | | | Maximum head-bill length | | | Maximum wing length | | |
| --- | --- | --- | --- | --- | --- | --- | --- | --- | --- | --- | --- | --- |
| Parameter | β ± SE | F_26,27_ | *P* | β ± SE | F_26,27_ | *P* | β ± SE | F_26,27_ | *P* | β ± SE | F_26,27_ | *P* |
| Intercept | -0.29 ± 0.22 |  |  | 36.02 ± 0.23 |  |  | 85.21 ± 0.31 |  |  | 239.61 ± 2.35 |  |  |
| Biologic parent feeding treatment^a^ | 0.32 ± 0.49 | 0.43 | 0.52 | 0.43 ± 0.51 | 0.71 | 0.41 | 0.21 ± 0.70 | 0.10 | 0.76 | 1.74 ± 5.05 | 0.12 | 0.73 |
| Hatching rank^b^ | -0.50 ± 0.51 | 0.96 | 0.33 | -0.05 ± 0.53 | 0.01 | 0.92 | -0.66 ± 0.72 | 0.85 | 0.37 | -7.62 ± 5.22 | 2.13 | 0.16 |
| Chick sex^c^ | **0.96 ± 0.46** | **4.83** | **0.04** | **0.94 ± 0.47** | **4.17** | **0.05** | **2.63 ± 0.64** | **15.60** | **0.001** | -1.44 ± 5.05 | 0.08 | 0.78 |
| Foster parent feeding treatment^a^ | 0.32 ± 0.49 | 0.45 | 0.51 | 0.49 ± 0.50 | 0.95 | 0.34 | 0.38 ± 0.69 | 0.31 | 0.59 | 0.18 ± 5.01 | 0.001 | 0.97 |
| Hatching date | **-1.01 ± 0.46** | **6.12** | **0.02** | -0.39 ± 0.55 | 0.51 | 0.48 | **-1.38 ± 0.64** | **5.34** | **0.03** | **-13.87 ± 4.78** | **9.97** | **0.004** |
| Sibling sex^c^ | -0.26 ± 0.48 | 0.29 | 0.59 | -0.16 ± 0.50 | 0.10 | 0.76 | 0.15 ± 0.68 | 0.05 | 0.83 | -5.41 ± 4.95 | 1.20 | 0.28 |
| Biologic parent feeding treatment^a^ × Hatching rank^b^ | 0.34 ± 1.00 | 0.12 | 0.73 | -1.62 ± 0.99 | 2.68 | 0.11 | 1.88 ± 1.38 | 1.85 | 0.19 | 15.45 ± 9.89 | 2.44 | 0.13 |

^a^ Relative to parents Fed before laying

^b^ Relative to chicks born from an A-egg

^c^ Relative to females

**Table S5.** Summary of the linear models describing variation in survival in senior chicks (N = 64) and junior chicks (N = 64), respectively. Significant terms (i.e. retained in the final model) are in bold type. β are the standardized parameter estimates (with their standard errors) taken prior to removal for terms not retained in the final model.

|  | Senior chicks | | | Junior chicks | | |
| --- | --- | --- | --- | --- | --- | --- |
| Parameter | β ± SE | χ²_1_ | P | β ± SE | χ²_1_ | P |
| Biologic parent feeding treatment^a^ | -0.74 ± 0.85 | 0.96 | 0.33 | 0.88 ± 0.50 | 3.44 | 0.063 |
| Hatching rank^b^ | 0.27 ± 0.81 | 0.13 | 0.72 | 0.59 ± 0.51 | 1.37 | 0.24 |
| Chick sex^c^ | **-1.49 ± 0.67** | **5.35** | **0.02** | -0.16 ± 0.44 | 0.13 | 0.72 |
| Foster parent feeding treatment^a^ | 0.01 ± 0.78 | 0.001 | 0.99 | -0.11 ± 0.45 | 0.06 | 0.81 |
| Hatching date | 0.18 ± 0.92 | 0.04 | 0.85 | -0.02 ± 0.47 | 0.003 | 0.95 |
| Sibling sex^c^ | 0.95 ± 0.82 | 1.40 | 0.24 | 0.60 ± 0.48 | 1.66 | 0.20 |
| Biologic parent feeding treatment^a^ × Hatching rank^b^ | _ | _ | _ | -0.62 ± 0.54 | 0.28 | 0.59 |

^a^ Relative to parents Fed before laying

^b^ Relative to chicks born from an A-egg

^c^ Relative to females

_ We did not for the interaction in senior chicks because the full model did not converge properly which led to poorly estimated estimates.
